# Primary Systemic Amyloidosis With Cardiac and Renal Involvement

**DOI:** 10.7759/cureus.25194

**Published:** 2022-05-21

**Authors:** Shin Ying Wong, Yen Shen Wong, Fatin Izni Nazri, Aisya Natasya Musa, Mohd Arif Mohd Zim

**Affiliations:** 1 Internal Medicine, Selayang Hospital, Selangor, MYS; 2 Internal Medicine, Universiti Teknologi MARA, Selangor, MYS; 3 Pathology, Selayang Hospital, Selangor, MYS

**Keywords:** nephrotic syndrome, cardiac failure, renal amyloidosis, pleural effusion, systemic amyloidosis

## Abstract

Systemic amyloidosis is a life-threatening disorder with a poor prognosis. Accurate and early diagnosis of the condition is of paramount importance as early initiation of therapy improves the prognosis and survival rate. A 49-year-old gentleman presented with recurrent right exudative pleural effusion. Thoracocentesis revealed unexplained exudative pleural effusion. Pleuroscopy and pleural biopsy showed chronic inflammatory changes with no atypical cells. Echocardiography revealed global dilated cardiomyopathy with an ejection fraction (EF) of 35%. He also had nephrotic range proteinuria of 2.83g/dL. A cystoscopy examination was performed due to macroscopic haematuria, and the bladder biopsy revealed focal acellular eosinophilic material within the stroma. Salmon red staining and apple-green birefringence were noticed under polarizing microscopy, suggestive of amyloidosis. Serum protein electrophoresis revealed raised alpha 1 globulin and alpha 2 globulins which support the diagnosis of primary systemic amyloidosis. Unfortunately, the patient passed away before the initiation of treatment due to cardiogenic shock. Early and less invasive tests for diagnosing systemic amyloidosis, such as abdominal fat pad aspiration and salivary gland biopsy, can be done. Given its systemic nature, early complications screening may benefit patients whereby targeted treatment can be given.

## Introduction

Extracellular deposition of protein fibrils causes a group of rare diseases called systemic amyloidosis. It is frequently underdiagnosed, thus portending a poorer prognosis due to the diagnostic dilemma. Deposition of amyloid protein can occur in the presence of abnormal proteins (e.g., light chain [AL] amyloidosis), excess of normal protein (e.g., reactive systemic [AA] Amyloidosis), and unknown causes (transthyretin [ATTR] amyloidosis) [1]. Amyloid deposition at any organ causes various clinical features, leading to difficulties and diagnosis delays [2]. We report a rare case of systemic amyloidosis presenting with recurrent unexplained pleural effusion, heart failure, and nephrotic range proteinuria posing diagnostic challenges. Understanding such a rare presentation of systemic amyloidosis will enhance the clinician's ability to make a prompt diagnosis.

## Case presentation

A 49-year-old gentleman presented with worsening shortness of breath, orthopnoea, and bilateral lower limb swelling for four months. He had no known medical illness. He was an ex-smoker with 30 pack-years smoking history. He also complained of dysphagia, hoarseness of voice, and weight loss of 10kg within three months. Clinical examination revealed reduced air entry over the right lower zone, raised JVP, and bilateral pedal edema. His serial plain chest radiograph showed recurrent right-sided pleural effusion, which became bilateral. Repeated thoracocenteses showed exudative effusion with negative culture. Pleuroscopy and pleural biopsy revealed chronic inflammatory changes with no atypical cells. Computed tomography of the thorax showed pulmonary edema and right middle and lower lobe collapse with an apparent filling defect within the left common venous pulmonary trunk and left ventricle. A serial echocardiogram showed a worsening ejection fraction from 65% to 35% within four months. The last echocardiogram also revealed a left atrial appendage clot. Biomarkers for heart failure, such as B-type natriuretic peptide (BNP), were raised at 7800 pg/ml. Urine complete examination and microscopic examination showed the presence of blood and protein of 2+, further examination with urine 24 hours protein showed nephrotic range proteinuria, urine protein of 2.83 g. Autoimmune antibodies such as the anti-neutrophil antibodies were negative. Investigations for tuberculosis and malignancy were negative.

**Figure 1:**
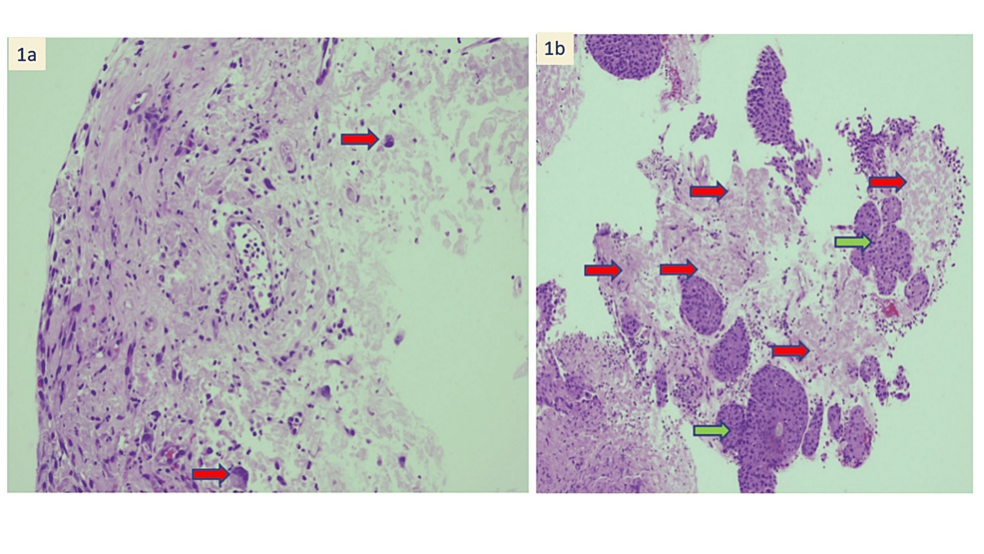
Bladder biopsy histopathological examination. (A) Mixed infiltrates of neutrophils and lymphocytes are seen within the stroma. Scattered stromal cells with reactive atypia are also noted (red arrow) (H&E stain, x 100 magnification). (B) Acellular eosinophilic amyloid deposition (red arrow) seen within the stroma. Green arrow show Von Brunn’s nests (H&E stain, x 100 magnification).

**Figure 2:**
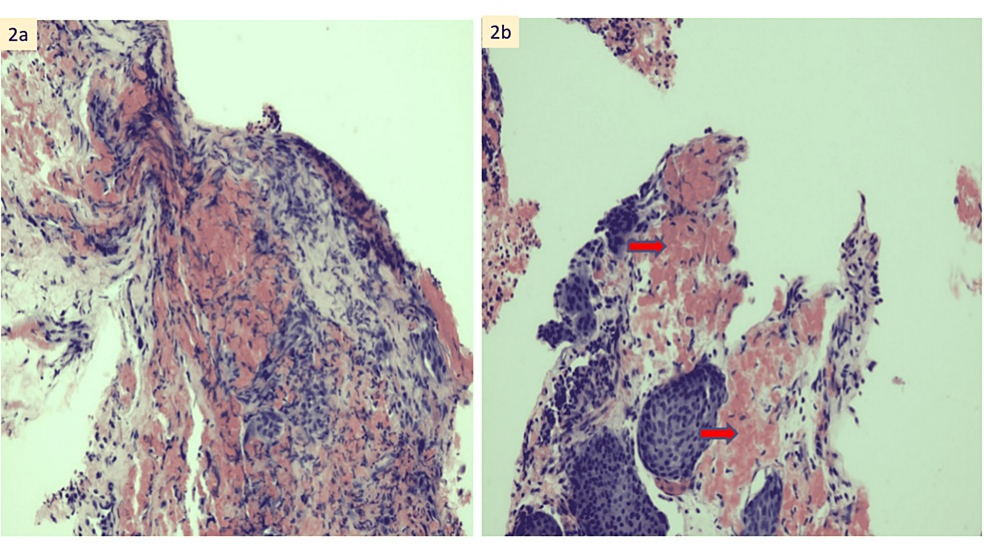
(A) Congo red stain, x 400 magnification. (B) Congo red shows salmon red staining of the amyloid (red arrow) (x 400 magnification)

During the hospital stay, he suffered from multiple complications from systemic amyloidosis. He developed a thromboembolic stroke in which computerized tomography (CT) brain showed recent multifocal infarction with gyriform hyperdensity in the right parietal-temporal region. There was also an episode of macroscopic haematuria after being started on an anticoagulant for intracardiac thrombus. A cystoscope examination was performed, and a bladder biopsy showed focal acellular eosinophilic material within the stroma [Figure 1]. The affected tissue showed salmon-red staining and apple-green birefringence under polarizing microscopy, pathognomonic for amyloidosis [Figure 2]. Serum protein electrophoresis results showed raised alpha 1 globulin and alpha 2 globulins. A diagnosis of primary systemic amyloidosis was suggested. Unfortunately, the patient passed away prior to initiation of treatment due to worsening cardiogenic shock.

## Discussion

Amyloidosis is a multisystemic disease characterized by the deposition of abnormal insoluble beta-sheet protein fibrils in different tissues. Amyloid deposition in different organs will lead to many clinical presentations, such as cardiomyopathy, hepatomegaly, proteinuria, macroglossia, autonomic dysfunction, ecchymoses, and recurrent pleural effusion [3].

Persistent pleural effusion secondary to amyloidosis is very rare. Due to its non-specific radiological and clinical findings, it isn't easy to diagnose accurately. However, a high clinical suspicion is needed to prevent delayed diagnosis and management. If history suggests multiorgan involvement, a pleural biopsy with congo red staining should be performed despite pleural effusion being transudative [4]. This persistent pleural effusion is most likely due to infiltration of amyloid into the parietal pleural surface, subsequently disrupting the balance between production and egression of the pleural fluid, causing persistent pleural effusion. Left atrial hypertension from cardiomyopathy may also contribute to the effusion. Untreated, these patients will have a median survival of only 1.6 months [5].

Aside from pleural effusion, cardiomyopathy is also one of the many complications suffered by our patients. Cardiac amyloidosis leads to restrictive infiltrative cardiomyopathy and heart failure, which may cause arrhythmias, embolic events, and sudden death [6]. On echocardiography, the appearance of "cherry‐like" apical sparing strain on two‐dimensional speckle‐tracking echocardiography based on Kumamoto criteria is associated with the increased left and right ventricular (LV, RV) thickness, atrial enlargement, restrictive LV filling pattern, and pericardial effusion raises suspicion for cardiac amyloidosis [7]. Nevertheless, the gold standard for diagnosing cardiac amyloidosis is via cardiac biopsy and advanced imaging using Proton emission tomography (PET) and cardiovascular magnetic resonance imaging (CMR). Cardiac medications are usually ineffective in this situation and may lead to hypotension, bradycardia, and worsening heart failure. Congestive heart failure accounts for about 40% of the death in these patients [8].

Dysphagia may happen due to vagus nerve neuropathy or amyloid myopathy. The oesophageal phase is affected due to achalasia-like movement as a result of reduced motility and increased rigidity of the musculature due to amyloid deposition [9]. Hoarseness of voice may also occur due to diffuse amyloid deposition or a single vocal cord nodule. The fragility of capillaries causing periorbital purpura due to vascular amyloidosis, which is pathognomonic of systemic amyloidosis, is seen in our patient. Swelling, alopecia, and glossitis may also be present [10].

Definitive diagnosis requires confirmation with congo red staining of biopsy specimens, such as the abdominal fat pad, salivary glands, and renal biopsy. It has been shown that abdominal fat pad biopsy has 81% sensitivity to detecting AL amyloidosis [11]. Plasma cell dyscrasia should be foremost ruled out due to the commonality of the condition and can be done via bone marrow biopsy, urine and serum electrophoresis, and serum immunoglobulin. Further investigations to determine other rarer types of amyloidosis such as mass spectrometry, immune electron microscopy, or immunohistochemistry can be done. 

There has been much recent progress in managing patients with systemic amyloidosis, involving aggressive measures to reduce the supply of amyloid precursor proteins and replacement of end-stage organ failure with dialysis and transplantation. The goal of treatment for AL amyloidosis is suppression or elimination of the B cell clone that produces immunoglobulins. This treatment involves cytotoxic drugs such as oral Mephalan, corticosteroids, and autologous stem cell transplantation [12].

## Conclusions

Great advancement in management and understanding of the pathophysiology of amyloidosis has been made to aid in their diagnosis. Improvements in diagnosis, treatment, organ replacement, and aggressive approaches to eliminating fibril precursors have revolutionized the clinical management of patients with systemic amyloidosis. Early diagnosis of amyloidosis remains an elusive goal that requires the education of both physicians and patients.
